# METTL3 suppresses anlotinib sensitivity by regulating m^6^A modification of FGFR3 in oral squamous cell carcinoma

**DOI:** 10.1186/s12935-022-02715-7

**Published:** 2022-09-27

**Authors:** Jie Chen, Shuai Li, Zhexun Huang, Congyuan Cao, Anxun Wang, Qianting He

**Affiliations:** 1 Department of Oral and Maxillofacial Surgery, The First Affiliated Hospital, Sun Yat-Sen University, Guangzhou, 510080 China; 2 Hospital of Stomatology, Guangdong Provincial Key Laboratory of Stomatology, Guanghua School of Stomatology, Sun Yat-Sen University, Guangzhou, 510055 China; 3 Department of Oral and Maxillofacial Surgery, College of Stomatology, Guangxi Medical University, Nanning, China; 4 Center of Oral Implantology, Stomatological Hospital, Southern Medical University, Guangzhou, 510280 China

**Keywords:** OSCC, Anlotinib, METTL3, FGFR3, TKI, m^6^A methylation

## Abstract

**Background:**

N6-methyladenosine (m^6^A) is an abundant nucleotide modification in mRNA, but there were few studies on its role in cancer drug sensitivity and resistance. Anlotinib has been proved to have effective antitumor effects in oral squamous cell carcinoma (OSCC) in our previous study. Here, we sought to investigate the treatment target of anlotinib and the function and mechanisms of m^6^A modification in regulating anlotinib effect in OSCC.

**Methods:**

Anlotinib treatment in a dose-dependent manner, western blotting, qRT-PCR and cell lost-of-function assays were used to study the treatment target of anlotinib in OSCC. RNA m^6^A dot blot assays, the m^6^A MeRIP-seq and MeRIP-qPCR, RNA and protein stability assays were used to explore the m^6^A modification of the treatment target of anlotinib. Cell lost-of-function assays after METTL3 depletion were conducted to investigate the effect of m^6^A modification level on the therapeutic effect of anlotinib in OSCC. Patient-derived tumor xenograft (PDX) models and immunohistochemistry staining were performed to study the relationship of METTL3 and antitumor sensitivity of anlotinib in vivo.

**Results:**

Anlotinib targeted FGFR3 in the treatment of OSCC and inhibited tumor cell proliferation and promoted apoptosis by inactivating the FGFR3/AKT/mTOR signaling pathway. METTL3 was identified to target and modify FGFR3 m^6^A methylation and then decrease the stability of mRNA. METTL3 expression level was related to the anlotinib sensitivity in OSCC cells in vitro and METTL3 knockdown promoted anlotinib sensitivity of OSCC cells by inhibiting the FGFR3 expression. PDX models samples furthermore showed that METTL3 and FGFR3 levels were tightly correlated with the anlotinib efficacy in OSCC.

**Conclusions:**

In summary, our work revealed that FGFR3 was served as the treatment target of anlotinib and METTL3-mediated FGFR3 m^6^A modification played a critical function in the anlotinib sensitivity in OSCC.

**Supplementary Information:**

The online version contains supplementary material available at 10.1186/s12935-022-02715-7.

## Background

Oral squamous cell carcinoma (OSCC) is the most common malignant tumor of the oral cavity and is prone to local recurrence and metastasis [1, 2]. To date, palliative drug therapy is an important treatment for patients with advanced, recurrent and metastatic OSCC [2], including traditional cytotoxic chemotherapy (Cisplatin, 5-FU and Paclitaxel), molecular targeted therapy (Cetuximab, Nimotuzumab and Sorafenib) and immunotherapy (Pembrolizumab and Nivolumab) [3–6]. However, tumors heterogeneity and the existence of drug resistance had been proved to limit the therapeutic effect of drug therapy. Thus, elucidation of mechanisms underlying the sensitivity and resistance to these drugs is needed to improve response in OSCC patients [6–8].

Anlotinib is a multi-target tyrosine kinase inhibitor (TKI) that can efficiently and selectively inhibit tyrosine kinase activity and then inhibit phosphorylation of downstream related proteins [9–11], such as vascular endothelial cell growth factor receptor 1, 2, 3 (VEGFR1, VEGFR2, VEGFR3), stem cell growth factor receptor (c-Kit), platelet-derived growth factor receptor beta (PDGFRβ) and fibroblast growth factor receptor (FGFR) 1–4. Multiple studies have shown that anlotinib has an anti-angiogenic and direct killing effect on a variety of tumor cells [11–15]. Our previous study also found that anlotinib monotherapy exerted favorable anticancer activity and manageable toxicities in patients with recurrent and metastatic OSCC [16]. However, the reported anlotinib insensitiveness and resistance impeded its anti-tumor effectiveness [17, 18].

Cancer therapeutic sensitivity is an intricated phenomenon affected by multiple mechanisms [19–22], including altered expression of drug influx/efflux transporters, the altered role of DNA repair and impairment of apoptosis, and altered epigenomics influencing upstream or downstream effectors. As a dynamic and reversible internal modification of RNA, N6-methyladenosine (m^6^A) methylation has been frequently reported promoting the tumor growth and metastasis in human cancers [23–28]. Targeting key regulators of m^6^A modification may be a potential cancer treatment target [23]. We previously studied that RNA m^6^A modification enzyme methyltransferase-like 3 (METTL3) promoted OSCC proliferation and metastasis through B cell-specific Moloney murine leukemia virus insertion site 1 (BMI1) m^6^A methylation [29]. To date, many studies have found that METTL3 regulated drug resistance in human cancers. Jin et al. [30] found that METTL3 induced RNA m^6^A modification by upregulating ATP binding cassette subfamily G member 2 (ABCG2) transcripts and regulated ABCG2-dependent multidrug resistance in non-small cell lung cancer. Uddin et al. [31] found that METTL3 depletion suppressed the expression of mutant p53 and sensitized colorectal cells to doxorubicin. METTL3 upregulates the expression of ubiquitin-conjugating enzyme E2B (UBE2B), a critical DNA damage repair enzyme, leading to drug resistance to 5-FU, cisplatin and gemcitabine [32, 33].

To date, the exact target and the underline mechanism of anlotinib in OSCC have not been sufficiently elucidated. In this study, we aimed to explore the exact target of anlotinib in OSCC and explore the role of m^6^A modification in anlotinib sensitivity of OSCC. Functional studies revealed that anlotinib targeted FGFR3 in the treatment of OSCC in vitro. m^6^A-RNA immunoprecipitation and sequencing (MeRIP-seq), MeRIP-qPCR, and RNA and protein stability assays revealed that METTL3 could modify FGFR3 mRNA m^6^A modification and decrease FGFR3 mRNA stability. METTL3 knockdown promoted anlotinib sensitivity of OSCC cells by increasing FGFR3 expression, and the expression level of METTL3 and FGFR3 was tightly correlated with the efficacy of anlotinib in OSCC.

## Methods

### Cell culture and transfection

Human normal oral epithelial keratinocytes (HOK) cells were purchased from ScienCell. Human umbilical cord veins cells (HUVECs), SCC9, SCC15, SCC25 and UM1 were purchased from ATCC. The OSCC cell lines were cultured in Dulbecco’s modified Eagle’s medium/F12 (Gibco, New York, USA) with 10% fetal bovine serum (FBS; Gibco) and 1% penicillin–streptomycin, incubated in 5% CO_2_ at 37℃. Anlotinib dihydrochloride was kindly provided by the Chia Tai Tianqing Pharmaceutical Group Co. Ltd. (Nanjing, China).

Lentivirus vectors containing METTL3 short hairpin RNA (shRNA) were purchased from OBiOc (Shanghai, China). Small interfering RNAs (siRNAs) (RiboBio, Guangzhou, China) targeting FGFR3 were designed to knockdown FGFR3 in OSCC. The indicated two shRNA and three siFGFR3 sequences are listed in Additional file 5: Table S1.

### Cell proliferation

The cells (5 × 10^3^) were planted in 96-well plates and then treated with specified concentrations of anlotinib for 24 h. Cell viability was performed using Cell Counting Kit-8 (CCK-8) (Dojindo, Japan) following the instructions. The formula was ([OD_t_—OD_b_] / [OD_c_—OD_b_]) × 100% (OD: optical density, t: treated sample, c: control sample, b: blank sample) [16]. Then IC_50_ values were counted with GraphPad Prism software (San Diego, CA, USA).

### Cell apoptosis assay

Cell apoptosis assays was performed with Annexin V-FITC/PI Apoptosis Assay Kit (MultiSciences, Hangzhou, China) according to the manufacturer’s protocol. After treatment with anlotinib for 24 h, flow cytometric analysis of fixed and stained cells was performed using CytoFLEX (Beckman Coulter, Brea, USA). Data analysis was performed using Flowjo software of 7.6 version (FLOWJO LLC, Ashland, USA).

### Western blotting

Total protein was lysed with proteinase inhibitor cocktail and radioimmunoprecipitation assay (RIPA) buffer with and conditionally adding phosphatase inhibitors (Sangon Biotech, Shanghai, China). The protein samples were then mixed with 5 × loading buffer, then denatured at 95℃ for 5 min, and electrotransferred to polyvinylidene fluoride (PVDF) membrane by 10% sodium dodecyl sulfate–polyacrylamide gel electrophoresis (SDS-PAGE). After blocking with 5% skim milk, the membrane was incubated in the primary antibody overnight at 4 °C, and then the secondary antibody diluted in Tris-buffered saline with Tween 20 (TBST) was used. Then, the protein-antibody complexes were detected using the enhanced chemiluminescence (ECL) on the Tanon 5200 Multi intelligent imaging system. The primary antibody used in this study are shown as below: VEGFR1 (Affinity, AF6204), VEGFR2 (Cell signaling Technology, 9698), VEGFR3 (Affinity, AF4201), FGFR1 (Signalway Antibody, 49,175), FGFR2 (Abcam, ab109372), FGFR3 (Abcam, ab133644), FGFR4 (Abcam, ab119378), c-Kit (Bioworld Technology, BS2433), PDGFRβ (Bioworld Technology, BS1764), GAPDH (Proteintech, 60,004), p-FGFR3 (Abcam, ab155960), AKT (Affinity, AF6261), p-AKT (Affinity, AF0016), mTOR (Affinity, AF6308), p-Mtor (Affinity, AF3308), BCL2 (Proteintech, 12,789–1-AP), BAX (Abcam, ab32503), METTL3 (Proteintech, 15,073–1-AP).

### Quantitative real-time PCR (qRT-PCR)

Total RNAs were extracted with TRIzol™ Reagent (Invitrogen, Carlsbad, USA) according to the manufacturer’s instruction. Reverse-transcription was conducted with 1 µg RNA using PrimeScript RT Master Mix kit (Takara, Japan). Then, quantitative PCR (qPCR) was carried out in StepOnePlusTM Real-Time PCR Instrument (Thermo Fisher Scientific) using the TB Green Premix Ex Taq II kit (Takara, Japan). The relative mRNA expression levels were calculated using β-Action as the internal control. All primer sequences are listed in Additional file 5: Table S1.

### MeRIP-seq and MeRIP-qPCR

The m^6^A MeRIP-seq and MeRIP-qPCR were performed according to the published procedures with slight modifications [29, 34]. Briefly, total RNA was fragmented with ZnCl2 and incubated with anti-m^6^A polyclonal antibody (Synaptic Systems, 202,003) or anti-IgG antibody (Abcam, ab150080) for 2 h at 4 °C. Then above RNA was incubated with protein A/G magnetic beads (Thermo Fisher Scientific; 88,802) for 2 h at 4 °C to obtain immunoprecipitated RNA fragments. The bound RNA was purified for sequencing library construction and qRT-PCR. The library was sequenced with the Illumina Next-Seq 500 sequencer and analyzed as previously described. Methylated sites on RNAs (peaks) were identified with MACS software.

### ***RNA m***^***6***^***A dot blot assays ***

After denaturing at 95 °C for 3 min, 2 μg of total RNA was crosslinked twice to the Hybond-N + membrane using a crosslinker with 1,200 μJ for 50 s. The membrane was incubated with m^6^A antibody, followed by secondary antibody. The dot blot signal intensity was detected by ECL with Tanon 5200 Multi intelligent imaging system. Then the membrane was stained with 0.02% Methylene blue (Sigma-Aldrich, St. Louis, USA) and scanned to reveal total input RNA content.

### RNA and protein stability assays

To assess the RNA and protein stability, RNA and protein decay assays were conducted. For RNA decay assay, OSCC cells were treated with actinomycin D (ActD, 5 μg/mL) for 0, 3, and 6 h. Total RNA was then isolated and the relative abundance of FGFR3 mRNA (relative to 0 h) was quantified using qRT-PCR. For protein decay assay, OSCC cells were treated with cycloheximide (CHX, 100 μg/mL) (Sigma-Aldrich) at 0, 2, 4, and 8 h. Then the expression of FGFR3 was detected via western blotting.

### Patient-derived tumor xenograft (PDX) models and immunohistochemistry staining (IHC)

The OSCC PDX models were established as previously described [16]. In brief, eight patients’ samples were used to establish the PDX models and the PDX mice were treated with 3 mg/kg anlotinib or normal saline (oral administration, once a day). The tumor growth inhibition (TGI) rate was calculated as (1-(Tn-T0)/(Cn-C0)) × 100% and presented in our previous study [16].

Tissue slides from PDX models were routinely deparaffinized and rehydrated. Following endogenous peroxidase quenching and antigen retrieval, the slides were blocked with 5% BSA for 30 min, followed by incubation with primary antibodies against METTL3 (Proteintech, 15,073–1-AP), FGFR3 (Signalway Antibody, 33,373) and p-FGFR3 (Affinity Biosciences, AF8439) overnight at 4℃. Then secondary antibodies and SABC were applied with SABC-POD (rabbit IgG) kit (BOSTER, Wuhan, China). The slides were stained using 3, 3’-diaminobenzidine (DAB) kit (BOSTER) and then counterstained with hematoxylin.

### Statistical analyses

The data were presented as the mean ± standard deviation (SD). Differences between the two groups were determined using the student’s *t*-tests. Statistical analyses were carried out using SPSS (version 25.0; IBM Corporation, Armonk, NY, USA). *P* < 0.05 were considered statistically significant. All experiments were performed in triplicate.

## Results

### Anlotinib targets FGFR3 and inhibits FGFR3 phosphorylation in the treatment of OSCC

**Fig. 1:**
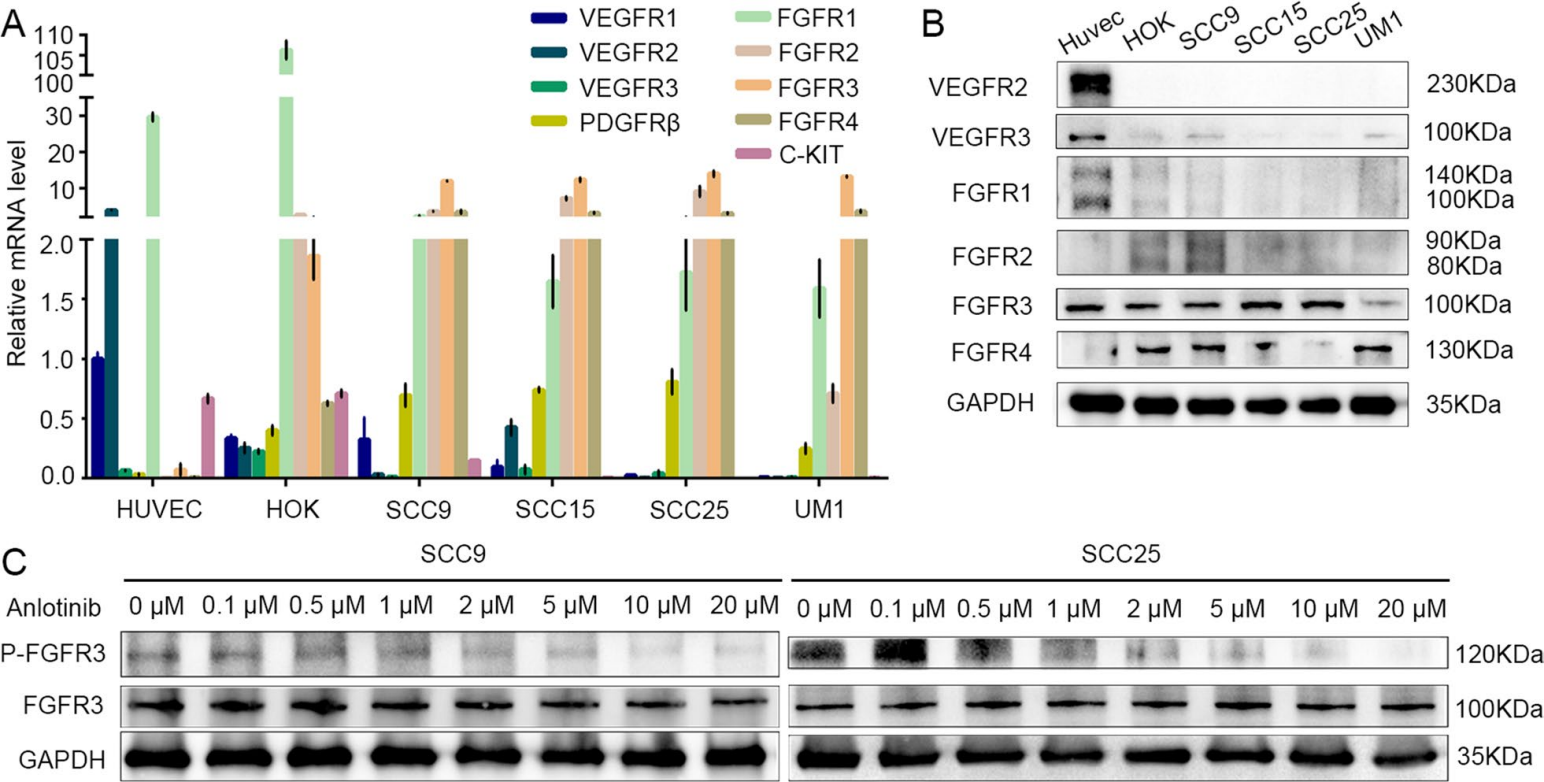
Anlotinib targets FGFR3 and inhibits FGFR3 phosphorylation in OSCC. **A** and **B** The tyrosine kinase inhibitor (TKI) targets of anlotinib were assessed by quantitative real-time PCR (qRT-PCR) and western blotting. **C** The expression and phosphorylation levels of FGFR3 were detected in concentration gradient anlotinib treated in SCC9 and SCC25 cells using the western blotting

To explore the possible anlotinib targets, firstly, we detected the mRNA expression levels of *VEGFR1-3, FGFR1-4, PDGFRβ* and *C-KIT* genes in four OSCC cell lines by qRT-PCR. We found that HUVECs mainly expressed *FGFR1 and VEGFR2* mRNA (Fig. 1A and Table 1). However, HOK and OSCC cells expressed low mRNA levels of *VEGFR1-3, PDGFRβ* and *C-KIT* genes, but with high mRNA levels of *FGFR1-4* genes (Fig. 1A and Table 1). Furthermore, four OSCC cell lines mainly expressed *FGFR3* mRNA among *FGFR1-4* (Fig. 1A and Table 1). We further evaluated the protein levels of the above genes by western blotting and found that VEGFR1, c-Kit and PDGFRβ were barely detected, VEGFR2 and VEGFR3 protein were basically not expressed in OSCC cells (Fig. 1B). Meanwhile, FGFR3 protein had the highest expression levels among FGFR1-4 protein in OSCC cells (Fig. 1B). Although the FGFR3 protein levels were not obviously changed after anlotinib treatment, we discovered the phosphorylation levels of FGFR3 significantly reduced in the anlotinib group in a dose-dependent manner in OSCC cells comparing with the non-anlotinib group (Fig. 1C and Additional file 1: Fig. S1). These above results implied that FGFR3 may act as a target of anlotinib in OSCC cells.

**Table 1:** Relative mRNA expression of therapeutic target of Anlotinib in OSCC cell lines

Cell line	VEGFR1	VEGFR2	VEFGFR3	PDGFRβ	FGFR1	FGFR2	FGFR3	FGFR4	c-KIT
HUVECs	1.000	3.896	0.065	0.034	29.590	0.002	0.073	0.008	0.667
HOK	0.335	0.255	0.226	0.401	106.328	2.551	1.861	0.628	0.709
SCC9	0.323	0.032	0.011	0.695	2.211	3.605	11.951	3.400	0.149
SCC15	0.095	0.426	0.074	0.740	1.648	7.160	12.289	3.144	0.005
SCC25	0.025	0.004	0.042	0.806	1.722	9.049	13.947	3.048	0.000
UM1	0.007	0.003	0.009	0.247	1.589	0.710	13.122	3.571	0.005

The VEGFR1 expression level of HUVEC was defined as 1

### FGFR3 expression level affects antitumor activity of anlotinib in OSCC

**Fig. 2:**
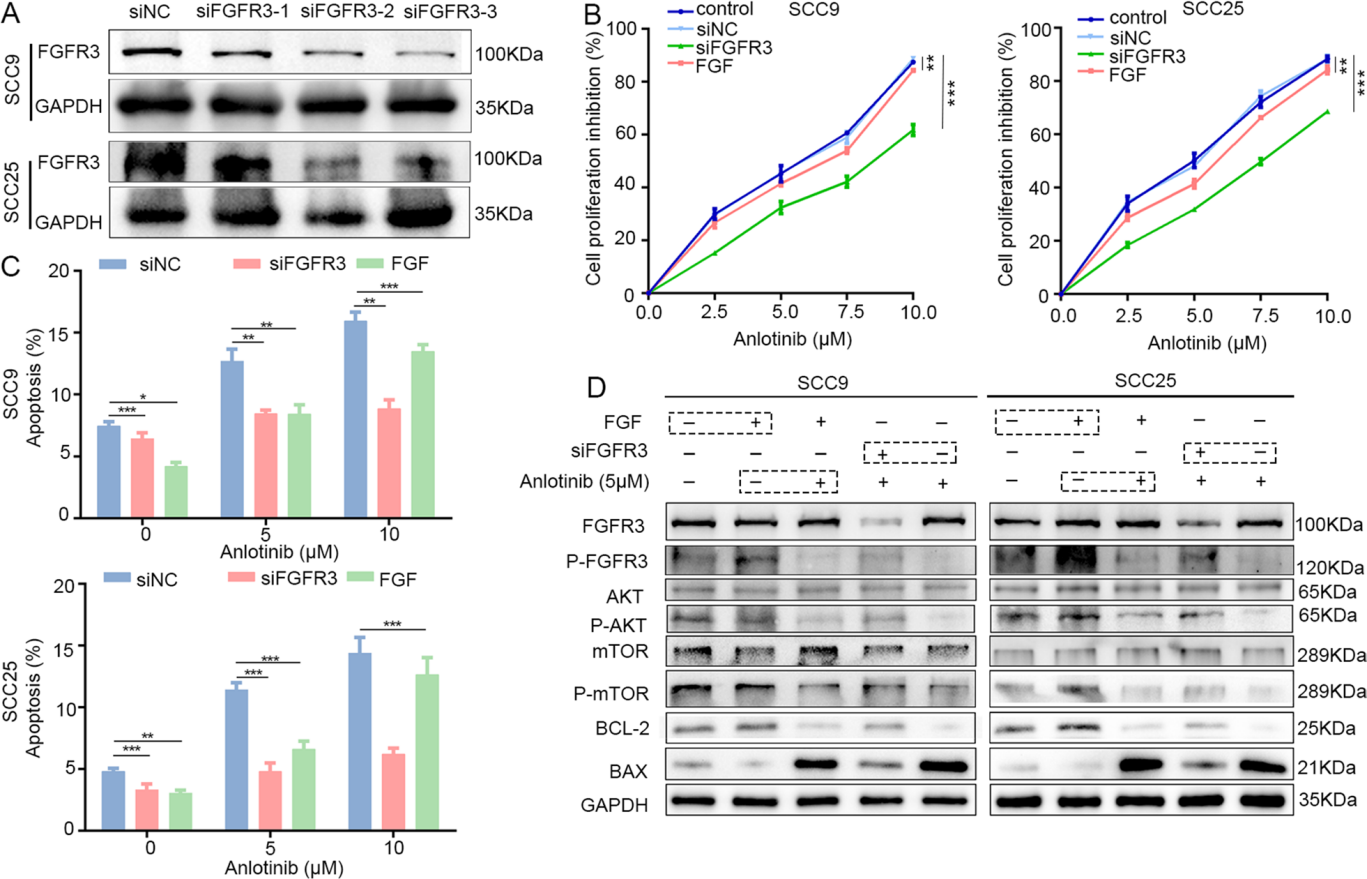
FGFR3 expression level affects antitumor activity of anlotinib in OSCC. **A** FGFR3 silenced effects were examined by western blotting. **B** Cell proliferation inhibition assay showed that the cytotoxic ability of anlotinib in OSCC cells (24 h) after transfected with siFGFR3 or treated with rhFGF. **C** Cell apoptosis assay showed the ratio of apoptosis cells of anlotinib-treated (24 h) OSCC cells after transfected with siFGFR3 or treated with rhFGF. **D** Western blotting was used to detect the expression of protein and phosphorylated protein of FGFR3, AKT and mTOR and apoptosis-related proteins in indicated treatment OSCC cells. *P < 0.05; **P < 0.01; ***P < 0.001

To further examine the role of FGFR3 in anlotinib-induced antitumor effects in OSCC, three different sequences of siFGFR3 were used to transfer into SCC9 and SCC15 cells, and the siRNA with the highest silencing efficacy (siFGFR3-3) was selected for subsequent experiments (Fig. 2A). As shown in Fig. 2B–D, anlotinib induced proliferation inhibition (Fig. 2B) and cellular apoptosis (Fig. 2C) in SCC9 and SCC25 in a dose-dependent manner. The phosphorylation of FGFR3, AKT and mTOR was decreased after anlotinib treatment (Fig. 2D). The ability of proliferation inhibition and apoptosis stimulation effects of anlotinib was significantly decreased after knockdown FGFR3 in OSCC cells, moreover, the phosphorylation levels of FGFR3, AKT and mTOR were slightly increased after knockdown FGFR3, suggesting that lower FGFR3 expression levels led to less anlotinib sensitivity of OSCC. Recombinant human FGF-2 (rhFGF-2, 20 ng/mL), which can stimulate autophosphorylation of FGFR3 and the phosphorylation levels of AKT and mTOR, also significantly diminished the ability of proliferation inhibition and apoptosis stimulation effects of anlotinib in OSCC cells (Fig. 2B, C and Additional file 2: Fig. S2). After anlotinib treatment, the phosphorylation levels of FGFR3, AKT and mTOR were decreased in rhFGF-2-treated plus anlotinib group. These changes caused subsequent corresponding changes of downstream apoptosis proteins, including BAX and BCL-2 (Fig. 2D).

Overall, these results suggested that anlotinib targeted and inhibited FGFR3 phosphorylation levels and then affected the subsequent AKT/mTOR phosphorylation to cause apoptosis of OSCC cells.

### ***METTL3 regulates FGFR3 mRNA m***^***6***^***A modification and decreases FGFR3 mRNA stability***

**Fig. 3:**
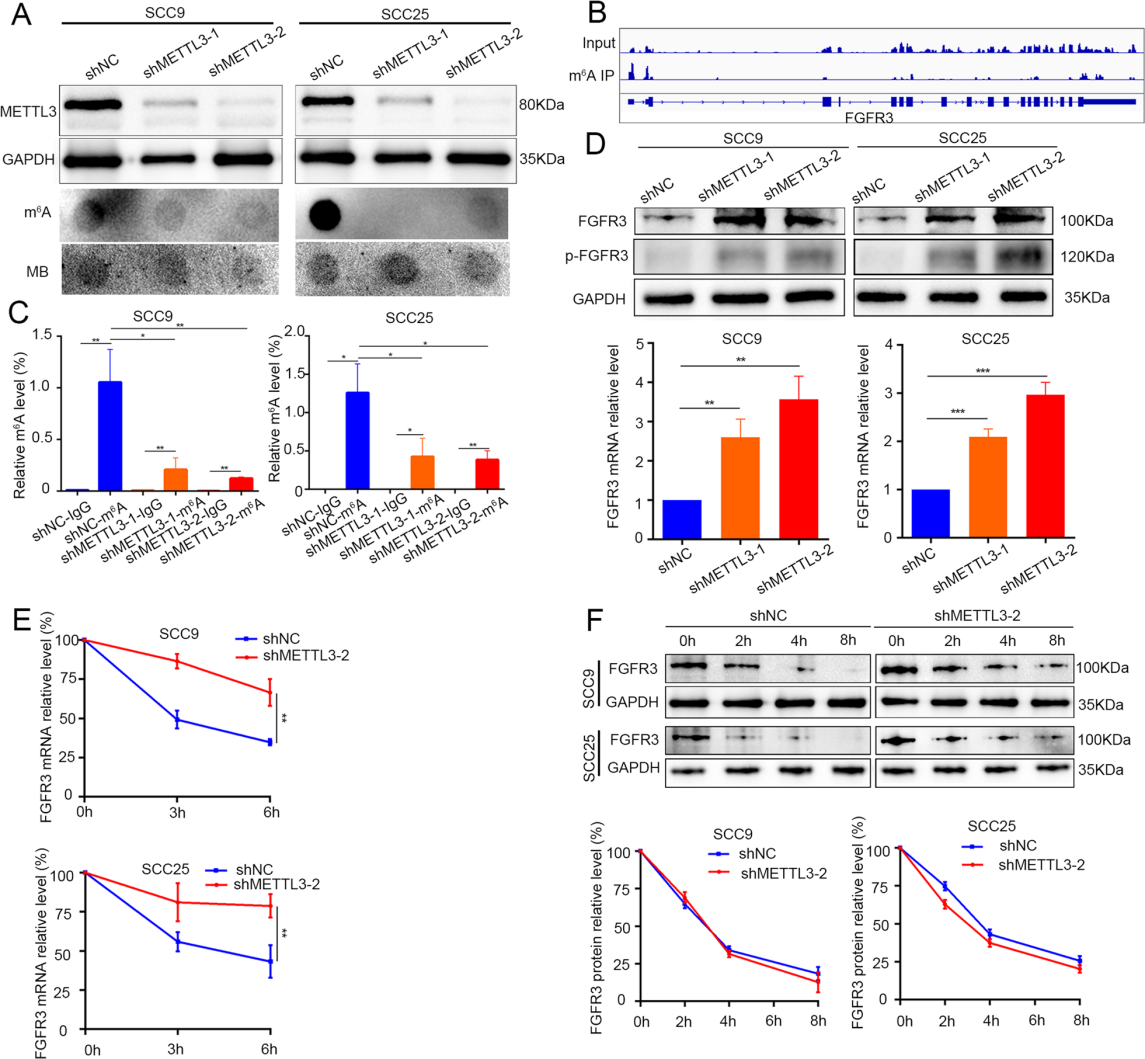
METTL3 regulates FGFR3 mRNA m^6^A modification and inhibits FGFR3 mRNA stability. **A** The METTL3 protein level and m^6^A level of RNA were detected by western blotting or dot blot in METTL3-knockdown OSCC cells (SCC9 and SCC25). **B** Representative m^6^A modification of FGFR3 in OSCC by MeRIP-seq (data from our previous study).**C** MeRIP-qPCR showed that relative FGFR3 m^6^A level was significantly decreased after METTL3 knockdown in SCC9 and SCC25 cells. **D** The FGFR3 protein and mRNA levels, and p-FGFR3 protein levels were significantly increased in METTL3 knockdown OSCC cells (SCC9 and SCC25). **E** FGFR3 mRNA stability was significantly decreased in METTL3-knockdown OSCC cells after treated with actinomycin D. F. FGFR protein stability was no significant changes between control cells and METTL3-knockdown OSCC cells by cycloheximide assay. Quantification of the protein optical density by ImageJ. *P < 0.05; **P < 0.01; ***P < 0.001

To investigate how METTL3 regulate FGFR3 in OSCC cells, firstly, we knocked down METTL3 in SCC9 and SCC25 cells and found that the m^6^A levels were significantly decreased in METTL3-knockdown group by dot-blot analysis (Fig. 3A). We then analyzed the data of m^6^A MeRIP-seq from our previous study [29] and found that the m^6^A peaks of FGFR3 were enriched near the 3’UTR regions (Fig. 3B). MeRIP-qPCR assay also showed that FGFR3 m^6^A modification was significantly decreased after METTL3 knockdown in SCC9 and SCC25 cells (Fig. 3C). Western blotting showed that METTL3 knockdown increased FGFR3 protein and mRNA expression levels, and also increased the FGFR3 phosphorylation levels (Fig. 3D). These data suggested that METTL3 might regulate FGFR3 expression at the post-transcriptional level.

Moreover, we compared FGFR3 mRNA and protein stability in METTL3-knockdown group with control cells by actinomycin D and cycloheximide assay. We determined that the stability of FGFR3 mRNA level was remarkably increased in METTL3-knockdown cells compared with control cells, but without changing the degradation rate of FGFR3 protein level (Fig. 3E, F). The results manifested that FGFR3 mRNA m^6^A modification by METTL3 could accelerate its degradation.

### METTL3 are inversely associated with anlotinib sensitivity in OSCC cells

**Fig. 4:**
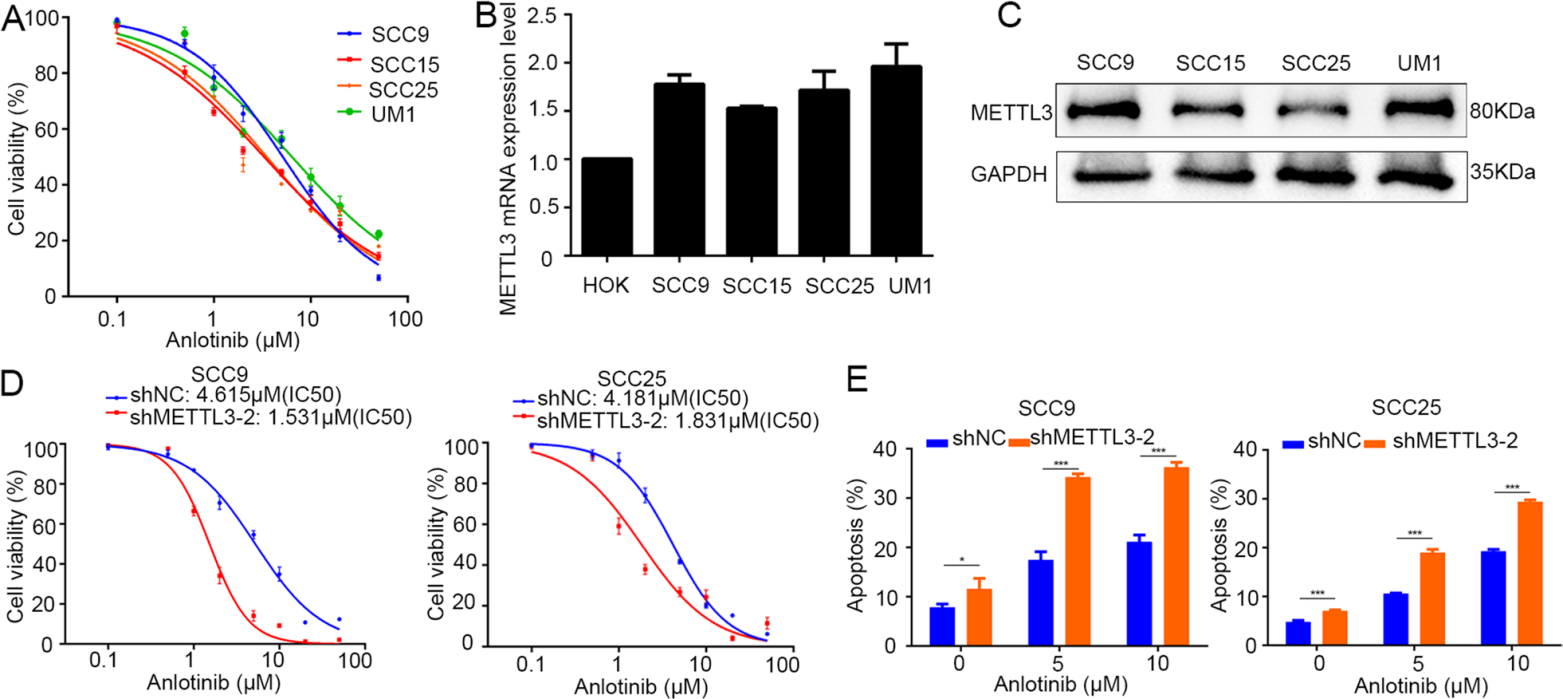
Levels of METTL3 are inversely associated with anlotinib sensitivity in OSCC cells. **A** The cytotoxic effect of anlotinib in different OSCC cell lines using cell viability assay. **B** and **C** The mRNA and protein levels of METTL3 in different OSCC cell lines using qRT-PCR and western blotting. **D** Cell viability assay showed the cytotoxic ability of anlotinib was significantly increased (decreased of IC50) in METTL3-knockdown OSCC cells compared with control cells (24 h). **E** Cell apoptosis assay showed the ratio of apoptosis cells in anlotinib-treated (24 h) cells was significantly increased after METTL3 knockdown in SCC9 and SCC25 cells lines. *P < 0.05; **P < 0.01; ***P < 0.001

To investigate the role of METTL3 in anlotinib treated OSCC cells, we explored the correlation between anlotinib sensitivity (IC50 values) and METTL3 expression level in OSCC cell lines. Four OSCC cell lines (SCC9, SCC15, SCC25 and UM1) were treated with anlotinib (Fig. 4A), and the expression of endogenous METTL3 was assessed by qRT-PCR and western blotting in OSCC cell lines (Fig. 4B, C). Strong correlation between IC50 and METTL3 expression level was identified in OSCC cells. The OSCC cells with higher METTL3 expression were less sensitive to anlotinib (higher IC50 values) (Table 2). METTL3 knockdown in OSCC cells sensitized the inhibitory effects of anlotinib in OSCC cells. The IC50 value decreased and the cellular apoptosis increased after anlotinib treatment in METTL3 knockdown OSCC cells (Fig. 4D, E and Additional file 3: Fig. S3).

**Table 2:** Correlation analysis of IC50 and METTL3 expression of different OSCC cells

Cell line	SCC9	SCC15	SCC25	UM1	Pearson
IC50	5.157	3.315	3.524	6.420	
METTL3 mRNA level	1.778	1.528	1.715	1.959	0.967
METTL3 protein level^a^	0.780	0.838	0.722	0.868	0.926

^a^The radio between the gray value of METTL3 protein and GAPDH protein. Pearson correlation coefficient was METTL3 expression level relative to IC50

### METTL3 affects the FGFR3 expression and antitumor efficacy of anlotinib in PDX models

**Fig. 5:**
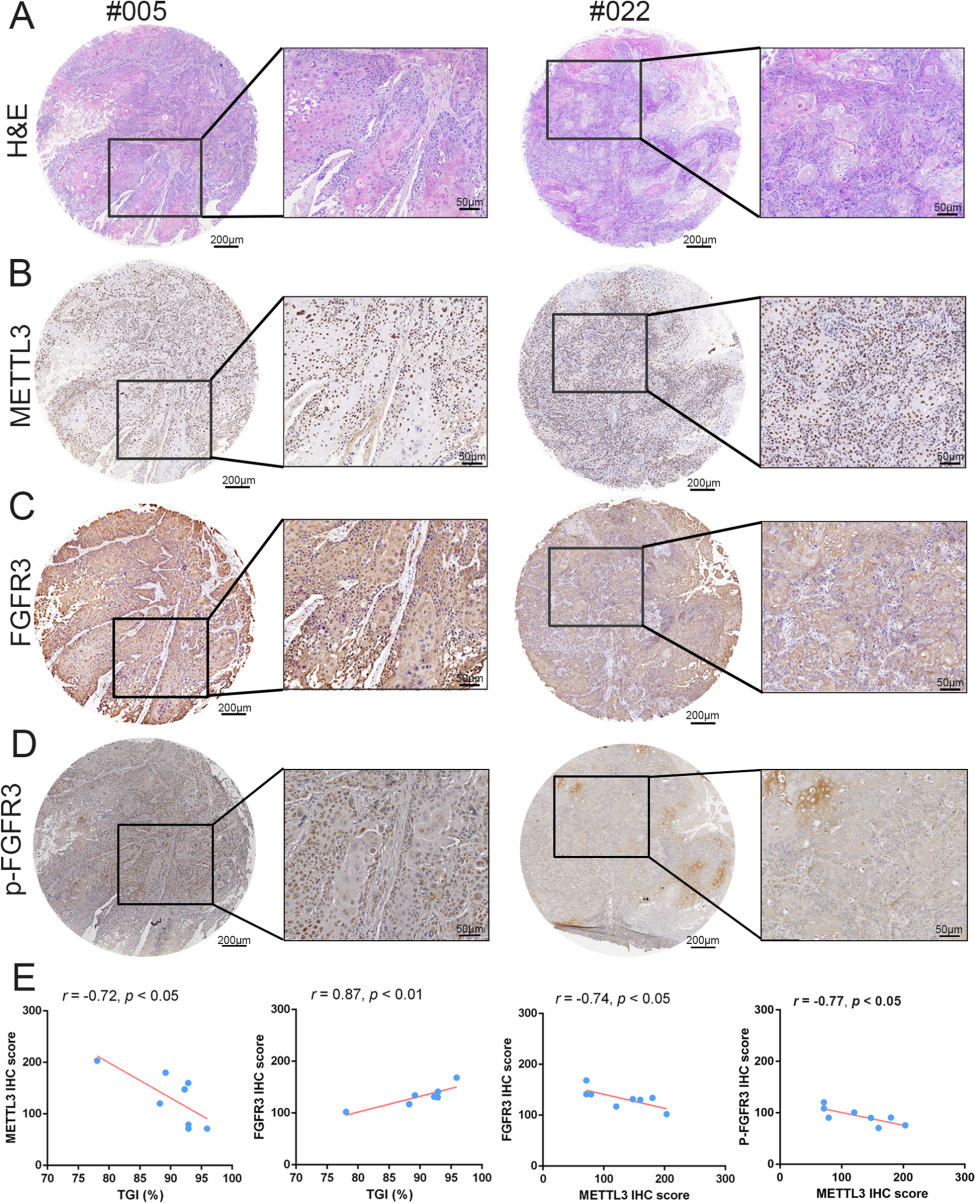
METTL3 affects the FGFR3 expression and antitumor efficacy of anlotinib in PDX models. **A**–**D** Representative H&E staining and IHC staining of METTL3, FGFR3 and p-FGFR3 in PDX models. #005 represented highest TGI rate (95.9%); #022 represented lowest TGI rate (78.07%). **E** The correlation between the IHC score of METTL3 and TGI rate, IHC score of FGFR3 and TGI rate, IHC score of METTL3 and FGFR3, IHC score of METTL3 and p-FGFR3

To verify the relationship between METTL3, FGFR3 and antitumor sensitivity of anlotinib in vivo, we explored the expression level of METTL3 and FGFR3 of tumor tissues from eight previously established OSCC PDX models [16]. After 30 days of anlotinib treatment, the TGI rate of anlotinib was evaluated to measure the antitumor sensitivity of anlotinib in OSCC PDX. TGI values in eight PDX models were 95.90 (#005), 92.28 (#010), 78.07 (#022), 92.85 (#024), 92.89 (#030), 92.89 (#032), 88.25 (#034) and 89.18% (#040). Histopathological examination (H&E) and IHC assay were used to detect the expression level of METTL3/FGFR3/p-FGFR3 in the control group of each PDX model (Fig. 5A–D). As shown in Fig. 5E, the expression level of METTL3/FGFR3 had significant correlation with TGI rate in PDX models. PDX models with the lower expression level of METTL3 or the higher expression level of FGFR3 displayed more sensitivity to anlotinib treatment with a higher TGI rate (Fig. 5E). Meanwhile, the expression levels of METTL3 and FGFR/p-FGFR3 were negatively correlated in each PDX sample significantly (Fig. 5E). Furthermore, we detected METTL3 and FGFR3 expression levels of 97 OSCC patients’ tissues by IHC, and the results verified that the expression levels of METTL3 and FGFR3 were negatively correlated significantly (Additional file 4: Fig. S4).

## Discussion

In recent decades, despite the application of various treatment modalities for OSCC, the five‐year overall survival rate of OSCC remains at 50% [35]. As a new, orally administered multi-target TKI, anlotinib exhibits excellent antitumor effects for several types of cancer [12, 36–38]. Our previous study [16] also confirmed that anlotinib exerted potent antiproliferation capability and induced apoptosis in OSCC cells, and favorable anticancer activity and manageable toxicities in patients with recurrent and metastatic OSCC. Until now tremendous studies have been performed to investigate the mechanism of anlotinib in the treatment of human cancers. Previous studies have found that PI3K/AKT/ mTOR signaling pathway and downstream apoptosis pathway were typical mechanism in anlotinib treatment [13]. Song et al. [13] found that anlotinib mainly inhibited the phosphorylation level of VEGFR2 and then affected PIK3/AKT signal activation in intrahepatic cholangiocarcinoma (ICC). Yang et al. [39] suggested that anlotinib suppressed cell proliferation and angiogenesis via inhibition of VEGFR-2/AKT and FGFR, PDGFRβ and their downstream signaling ERK in colorectal cancer. In this study, we further confirmed that the antitumor effect of anlotinib was conducted by targeting FGFR3 and inhibiting the phosphorylation level of FGFR3, and subsequent inhibition of the AKT/mTOR and apoptosis signaling pathway in OSCC. Taken together, these results suggested that anlotinib may be involved in the FGFR3/AKT/mTOR signaling pathway in the therapeutics of OSCC.

As the most pervasive internal modification of mRNA, m^6^A modification is installed by a methyltransferase complex (e.g., METTL3-METTL14), erased by demethylases (e.g., FTO and ALKBH5), and can be recognized by readers (e.g., YTHDF1-3, IGF2BP1-3) [29]. Studies have proved that mRNA m^6^A modification can affect RNA splicing, RNA stability, RNA translation efficiency, RNA secondary structure and RNA subcellular localization [23]. Tremendous studies have shown that METTL3 promotes tumor growth, metastasis, and drug resistance in human cancers [26–28, 40, 41]. Recently, Yan et al. [42] identified the dynamic m^6^A methylome as an additional epigenetic driver for reversible TKI tolerance. Ianniello et al. [43] also revealed that downregulation of METTL3 and METTL14 overcame the resistance of chronic myeloid leukemia cells to the TKI imatinib mesylate (imatinib) through regulating ribosome levels and translation. Sa et al. [44] demonstrated that insulin-like growth factor 2 mRNA-binding protein 2 (IGF2BP2)-dependent ERBB2 signaling activation contributes to acquired resistance to TKI of radioiodine-refractory papillary thyroid cancer. In the present study, we observed the function of m^6^A methylation in regulating anlotinib sensitivity of OSCC, providing a mechanistic paradigm for drug sensitivity in cancer. Cell lost-of-functional assays revealed that FGFR3 act as the anlotinib target in OSCC cells. Our MeRIP-seq and MeRIP-qPCR results demonstrated that FGFR3 was selectively m^6^A modified in OSCC. Depletion of METTL3 decreased FGFR3 m^6^A methylation and mRNA stability, and promoted anlotinib sensitivity of OSCC cells. OSCC PDX models verified that METTL3 and FGFR3 levels were tightly correlated with the anlotinib efficacy in the treatment of OSCC, also the expression levels of METTL3 and FGFR were significantly negatively correlated in each PDX sample. Thus, METTL3-mediated m^6^A modification played a critical function in the anlotinib sensitivity of OSCC.

## Conclusions

Anlotinib targeted and inhibited FGFR3 phosphorylation in the treatment of OSCC. METTL3 modified FGFR3 m^6^A methylation and decreased its stability which attenuated the effect of anlotinib. The METTL3/m^6^A axis could serve as a biomarker to predict response of the OSCC patients to the anlotinib treatment.

## Supplementary Information

**Additional file 1: Figure S1.** Quantification of the expression and phosphorylation levels of FGFR3 after anlotinib treated in OSCC cells. (corresponded to Fig. 1C).

**Additional file 2: Figure S2.** Representative images of cell apoptosis assays showed the ratio of apoptosis cells of anlotinib-treated (24h) in OSCC cells. (corresponded to Fig. 2C).

**Additional file 3: Figure S3.** Representative images of cell apoptosis assays showed the ratio of apoptosis cells of anlotinib-treated (24h) after METTL3 knockdown in OSCC cells. (corresponded to Fig. 4E).

**Additional file 4: Figure S4.** Representative images of IHC staining of METTL3 and FGFR3 of OSCC patients’ tissues, and the correlation between IHC score of METTL3 and FGFR3. (corresponded to Fig. 5).

**Additional file 5: Table S1.** The shRNA or siRNA sequence and primer sequence for RT-PCR.

## Data Availability

All data generated or analyzed during this study are included in this article [and its additional files].

## Abbreviations

m^6^A: N6-methyladenosine
OSCC: Oral squamous cell carcinoma
PDX: Patient-derived tumor xenograft
TKI: Tyrosine kinase inhibitor
VEGFR: Vascular endothelial cell growth factor receptor
c-Kit: Stem cell growth factor receptor
PDGFRβ: Platelet-derived growth factor receptor beta
FGFR: Fibroblast growth factor receptor
METTL3: Methyltransferase-like 3
BMI1: B cell-specific Moloney murine leukemia virus insertion site 1
ABCG2: ATP binding cassette subfamily G member 2
UBE2B: Ubiquitin-conjugating enzyme E2B
MeRIP-seq: M^6^A-RNA immunoprecipitation and sequencing
HOK: Human normal oral epithelial keratinocytes
HUVECs: Human umbilical cord veins cells
FBS: Fetal bovine serum
siRNA: Small interfering RNA
shRNA: Short hairpin RNA
TGI: Tumor growth inhibition
