# Supplementary figures and images for: METTL3 suppresses anlotinib sensitivity by regulating m6A modification of FGFR3 in oral squamous cell carcinoma
Source: Cancer Cell Int. 2022 Sep 27;22:295. doi: 10.1186/s12935-022-02715-7 (PMC9516809; doi:10.1186/s12935-022-02715-7)

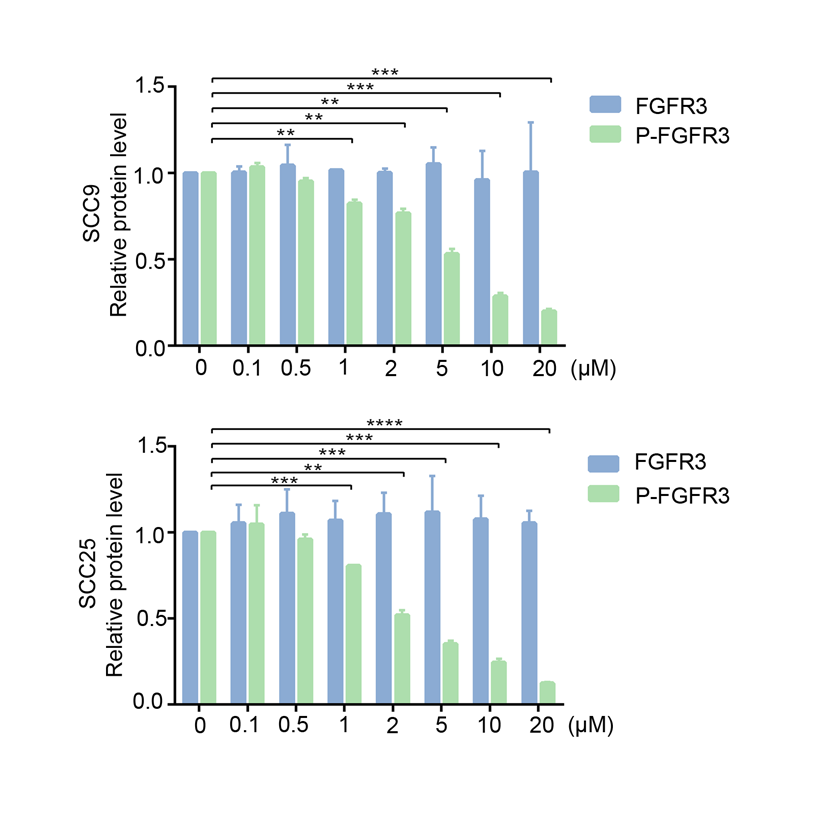

Supplement: Supplementary file 1 — Additional file 1: Figure S1. Quantification of the expression and phosphorylation levels of FGFR3 after anlotinib treated in OSCC cells. (corresponded to Fig. 1C). [file 12935_2022_2715_MOESM1_ESM.tif]

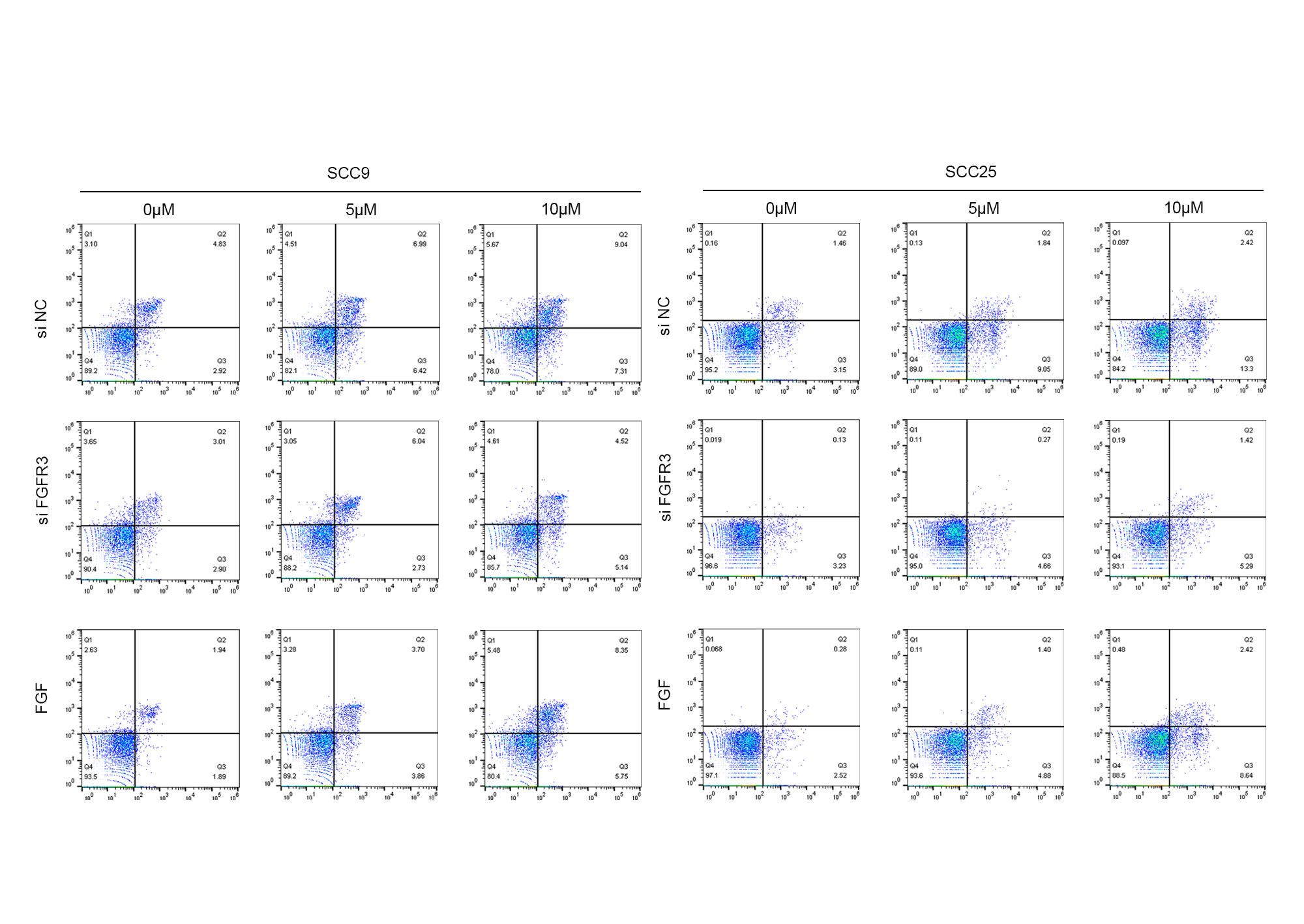

Supplement: Supplementary file 2 — Additional file 2: Figure S2. Representative images of cell apoptosis assays showed the ratio of apoptosis cells of anlotinib-treated (24h) in OSCC cells. (corresponded to Fig. 2C). [file 12935_2022_2715_MOESM2_ESM.tif]

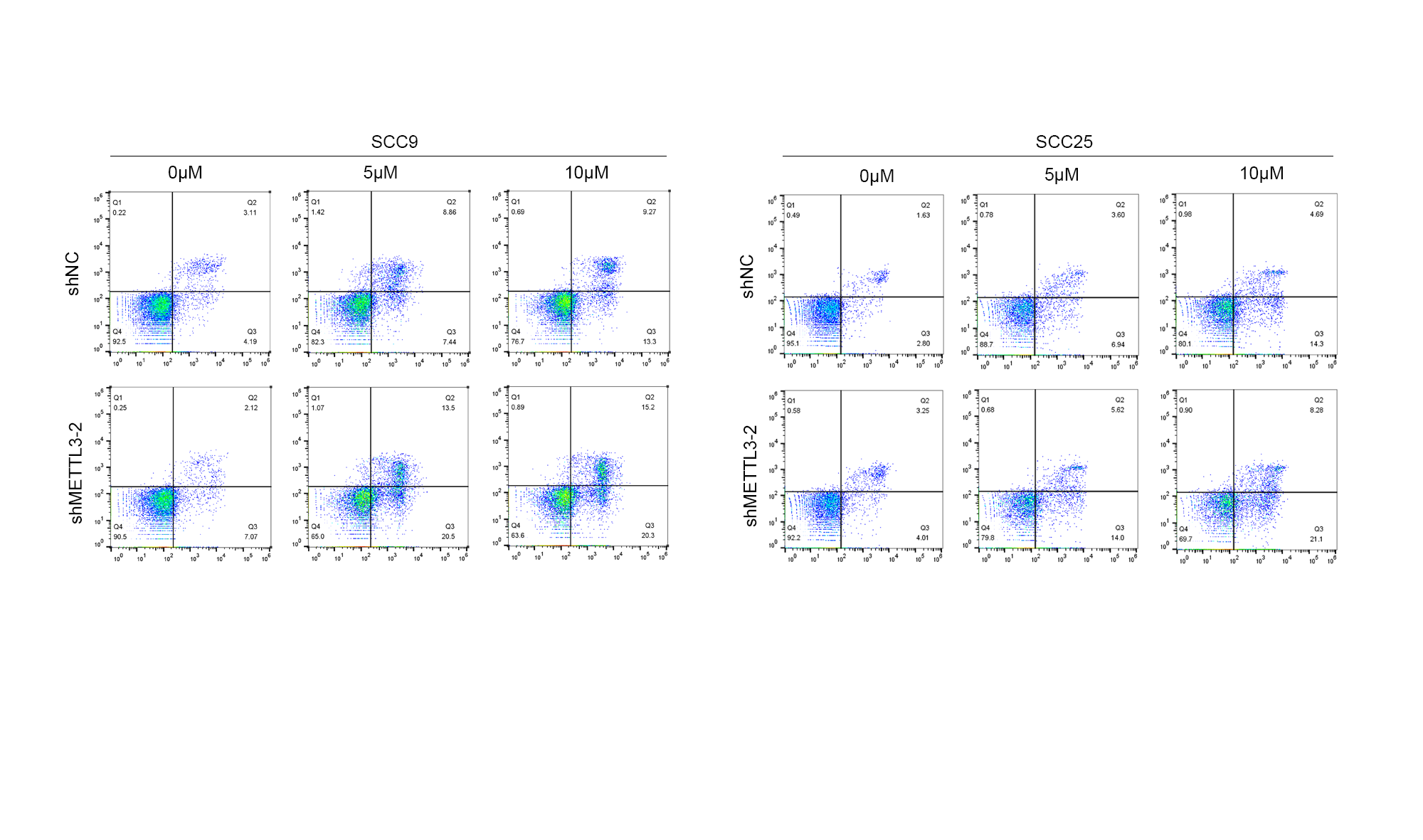

Supplement: Supplementary file 3 — Additional file 3: Figure S3. Representative images of cell apoptosis assays showed the ratio of apoptosis cells of anlotinib-treated (24h) after METTL3 knockdown in OSCC cells. (corresponded to Fig. 4E). [file 12935_2022_2715_MOESM3_ESM.tif]

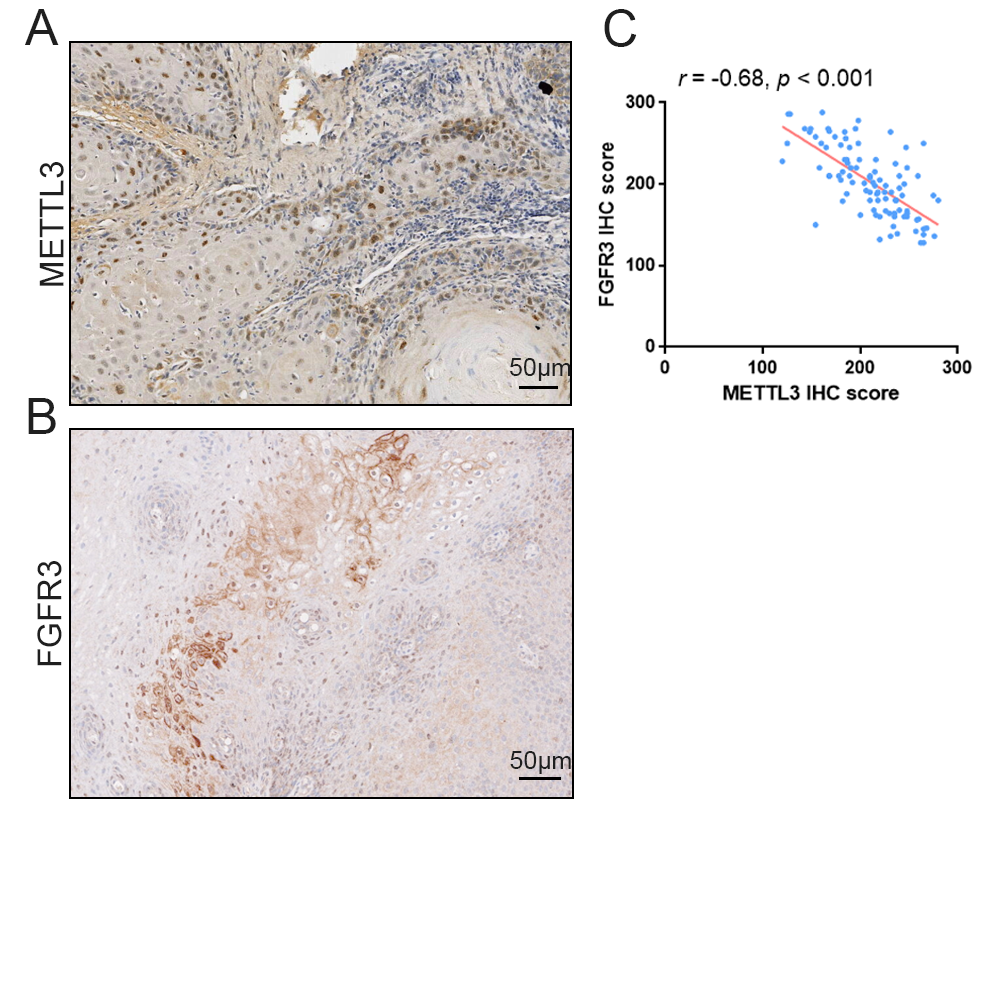

Supplement: Supplementary file 4 — Additional file 4: Figure S4. Representative images of IHC staining of METTL3 and FGFR3 of OSCC patients’ tissues, and the correlation between IHC score of METTL3 and FGFR3. (corresponded to Fig. 5). [file 12935_2022_2715_MOESM4_ESM.tif]
