# Supplementary material for: METTL3 suppresses anlotinib sensitivity by regulating m6A modification of FGFR3 in oral squamous cell carcinoma
Source: Cancer Cell Int. 2022 Sep 27;22:295. doi: 10.1186/s12935-022-02715-7 (PMC9516809; doi:10.1186/s12935-022-02715-7)
Supplement: Supplementary file 5 — Additional file 5: Table S1. The shRNA or siRNA sequence and primer sequence for RT-PCR. [file 12935_2022_2715_MOESM5_ESM.docx]

**Table S1: The shRNA or siRNA sequence and primer sequence for RT-PCR**

| Oligonucleotides |  |
| --- | --- |
| shRNA targeting sequence: METTL3 #1 | GCTGCACTTCAGACGAATT |
| shRNA targeting sequence: METTL3 #2 | GCTCAACATACCCGTACTA |
| siRNA targeting sequence: FGFR3 #1 | GAGGAAAAGGCUGGUACAA |
| siRNA targeting sequence: FGFR3 #2 | CACAUGUCCAGCACCUUGU |
| siRNA targeting sequence: FGFR3 #3 | GAUGCUGUGUAUAUGGUAU |
| Primer targeting human FGFR3 | F: 5’-TCCATCTCCTGGCTGAAGAACG-3’  R: 5’-TGTTCTCCACGACGCAGGTGTA-3’ |
| Primer targeting human c-KIT | F: 5’- CACCGAAGGAGGCACTTACACA -3’  R: 5’- TGCCATTCACGAGCCTGTCGTA -3’ |
| Primer targeting human VEGFR1 | F: 5’- CCTGCAAGATTCAGGCACCTATG -3’  R: 5’- GTTTCGCAGGAGGTATGGTGCT -3’ |
| Primer targeting human VEGFR2 | F: 5’- GGAACCTCACTATCCGCAGAGT -3’  R: 5’- CCAAGTTCGTCTTTTCCTGGGC -3’ |
| Primer targeting human VEGFR3 | F: 5’- TGCGAATACCTGTCCTACGATGC -3’  R: 5’- CTTGTGGATGCCGAAAGCGGAG -3’ |
| Primer targeting human PDGFRβ | F: 5’- TGCAGACATCGAGTCCTCCAAC -3’  R: 5’- GCTTAGCACTGGAGACTCGTTG -3’ |
| Primer targeting human FGFR1 | F: 5’- GCACATCCAGTGGCTAAAGCAC -3’  R: 5’- AGCACCTCCATCTCTTTGTCGG -3’ |
| Primer targeting human FGFR2 | F: 5’- GTGCCGAATGAAGAACACGACC -3’  R: 5’- GGCGTGTTGTTATCCTCACCAG -3’ |
| Primer targeting human FGFR4 | F: 5’- AACACCGTCAAGTTCCGCTGTC -3’  R: 5’- CATCACGAGACTCCAGTGCTGA -3’ |
